# Solvent Redistribution Method To Determine Solubility and Aggregation: High Throughput, Accuracy, and Sustainability

**DOI:** 10.1021/acs.jpcb.5c03073

**Published:** 2025-08-19

**Authors:** O. B. Tarun, N. Dupertuis, David. M. Wilkins, S. Roke

**Affiliations:** † Laboratory for fundamental BioPhotonics, Institute of Bioengineering (IBI), School of Engineering (STI), 27218 École Polytechnique Fédérale de Lausanne (EPFL), CH-1015 Lausanne, Switzerland; ‡ Centre for Quantum Materials and Technologies, School of Mathematics and Physics, 1596 Queen’s University Belfast, Belfast BT7 1NN, Northern Ireland U.K.; § Institute of Materials Science and Engineering (IMX), School of Engineering (STI), École Polytechnique Fédérale de Lausanne (EPFL), CH-1015 Lausanne, Switzerland

## Abstract

The pharmaceutical industry is responsible for 4.4% of global CO_2_ emissions. Assays used in drug discovery and development are major contributors to waste, including solubility measurements. These are either accurate but slow and energy-intensive (HPLC, centrifugation, filtration), contributing significantly to global CO_2_ emissions, or fast and economical but inaccurate (turbidity/nephelometry). Here, we exploit the sensitivity of high-throughput angle-resolved second harmonic scattering to detect nanoscale interfacial fluctuations in the solvent around the solute. Classical nucleation theory and nonlinear light scattering modeling show that, prior to aggregation, the solvent interfacial area changes drastically. This leads to changes in the standard deviation of the intensity at specific scattering angles, which can be used to obtain insights into the solubilizing mechanism. Exploiting the coherent nature of the emission, the detection limit for solubility/aggregation is reduced from ∼1 μM to ∼1 nM, achieving over a thousandfold sustainability gain, potentially permitting a reduction in the pharmaceutical industry’s global CO_2_ emissions by ∼3.5%.

## Graphical abstract

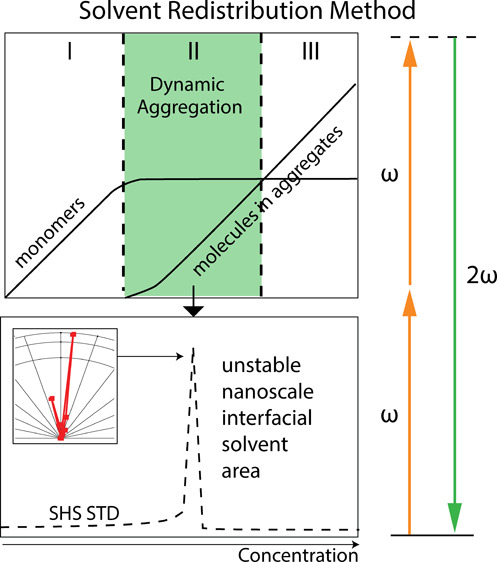

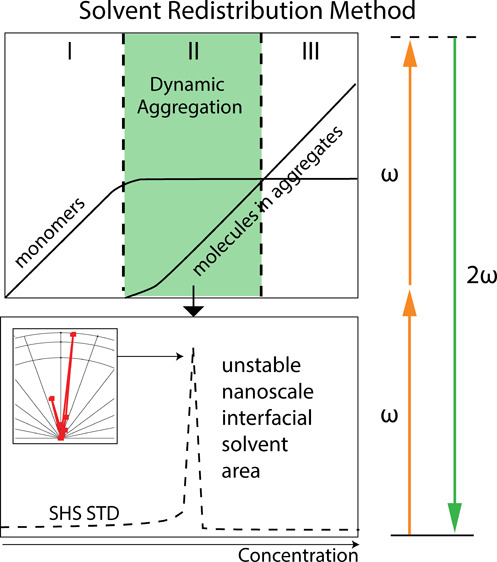

## Introduction

The solubility of a substance is defined as the maximum amount that can be dissolved in a solvent before aggregates form.^1^ In solution, molecules redistribute statistically. They can aggregate into dimers, trimers, or larger objects, and these objects can also split up again into monomers. This growth and decay process results in aggregation when the aggregates are thermodynamically more stable than the monomers, as illustrated in Figure 1A. Region I is the stable monomer regime, region III is the stable aggregate regime, and region II represents an unstable transition region where statistical fluctuations dominate.^2^ While the macromolecular thermodynamics of aggregation is well understood,^1^ its kinetics and molecular level dynamics are not, as they require technology that can continuously measure the molecular structures of solvent and solute in a dynamically fluctuating environment.

**Figure 1:**
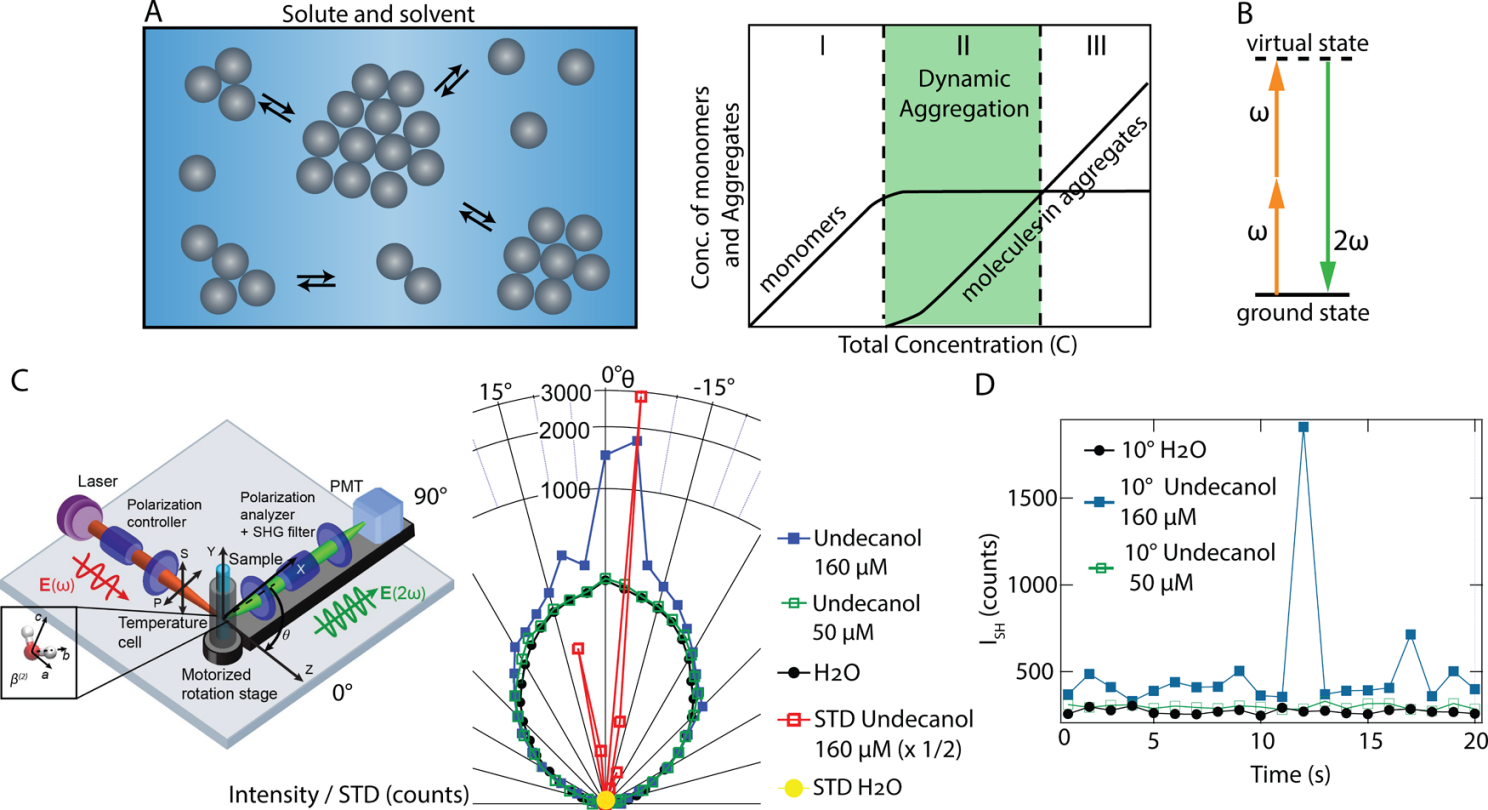
Coherent intensity fluctuations are linked to solubility and aggregation. (A) Illustration of classical thermodynamic aggregation processes, where solutes can statistically aggregate into dimers, trimers, and larger aggregates. Region I is the stable monomer region, region III is the stable aggregate region, and region II is a transition region where dynamic aggregation and statistical fluctuations dominate. (B) Illustration of nonresonant second harmonic generation where two incident photons with frequency ω combine into an SH photon at 2ω. (C) Sketch of the angle-resolved second harmonic scattering experiment and polar plot of the average SH intensities of pure water (black solid circles) and 50 and 160 μM solutions of undecanol in water (green open squares and blue solid squares). The polar plot also shows the standard deviations (STDs) of pure water and 50 μM undecanol in water solutions (yellow circles, data points overlap) and that of a solution of 160 μM undecanol in water. (D) Time-trace measurement from Figure 1C (*N* = 20 measurements, 1 s integration time) at θ = 10°.

Besides being of fundamental interest, solubility is also a parameter that is needed for nearly every chemical experiment and therefore plays an important role in the R&D programs of the chemical and pharmaceutical industries. In the formulation phase of drug development, solubility is a key parameter that needs to be optimized to increase the efficacy of drugs.^3–5^ The discovery and development of a single drug requires >10,000 solubility measurements and wastes >16 million metric tons of chemicals.^5,6^ Ideally, such measurements require a combination of high sensitivity, high throughput, and minimal use of chemicals and compounds. However, current state-of-the-art solubility measurements face two major drawbacks: they consume enormous amounts of energy and chemicals,^3,5,7–9^ and are relatively insensitive. At these costs, only a modest measurement throughput is possible, with typically 2000–5000 compounds per year per instrument,^5^ at a ∼1 μM sensitivity.^9^ With the increasing scale of drug discovery, and the increased use of nanotechnology, for which hundreds of thousands of compounds are synthesized,^10,11^ there is no practical, accurate, and sustainable solubility measurement technique available to support it.^12^

Solubility is commonly measured by either first separating aggregates from the main phase, followed by detection, or by detecting aggregates directly.^5,7–9^ Separation involves either filtration or high-performance liquid chromatography (HPLC). The separated compounds are then measured using Ultraviolet–visible (UV–vis) spectroscopy or mass spectrometry. This separation step requires significant amounts of solute and solvent, generating a lot of waste. Furthermore, the measured solubility value is strongly affected by the chosen method of filtration and separation steps. Although the method to detect what is dissolved (through mass spectrometry or UV–vis) is sensitive, the complexity of the measurement with calibration, filtration, and separation steps contributes significantly to the variability of the measurement.^13,14^ Direct measurements make use of linear light scattering, where the turbidity or cloudiness of the solution is recorded.^9,15^ In this case, higher detection limits are achieved, albeit with a smaller waste of chemicals. Turbidity measurements record the loss of transmitted light that occurs during light scattering. The sensitivity to solubility is determined by how effectively light is scattered by aggregates, which is determined by the difference in refractive index between the solute aggregates and the solvent. This depends on the volume of the solute in aggregated form and is therefore relatively insensitive, especially for small molecules. Because a change in light scattering can also be due to intrinsic fluctuations in the sample, the sensitivity/detection limit levels off and cannot be further optimized^16^ below ∼20 μM.^9^ Chemical specificity can be increased—at the cost of reducing the sensitivity to >1 mM—if spectroscopic methods such as Raman scattering in combination with multivariate curve resolution methods are employed. This allows for statements to be made on the type of aggregates.^17^ Second harmonic scattering (SHS), a process in which two near-infrared photons of frequency ω combine into a second harmonic (SH) photon of frequency 2ω,^18^ has also been used to measure solubility. Aggregation/micellization of fluorophores has been explored by using resonant enhancement to increase the sensitivity. This approach is generally limited to specific fluorophores.^19^ Nonresonant SH generation (Figure 1B) is also possible. Isotropic materials do not emit coherent SH photons under the electric dipole approximation.^20^ Noncentrosymmetric materials do, and this has enabled the sensitive detection of protein crystallization,^21,22^ to follow the kinetics of the growth of metal–organic framework particles,^23^ and to track harmonophores for tumor detection.^24^ Recently, nonresonant SHS has been used to detect micellization of surfactants, whereby the hyperpolarizability of the surfactant and its aggregates was detected at a 90° scattering angle.^25^ The detection limit for this method is ∼10 mM, as it is limited by the weak nonlinear optical conversion efficiency.

Here, we present the solvent redistribution (SR) method, a high-throughput and sustainable approach to measure solubility. High-throughput angle-resolved SHS^26,27^ (Figure 1C) is used to detect interfacial fluctuations in the solvent that surrounds the solute. The scattering angle-dependent, background-free SH emission and intrinsic statistical fluctuations in the sample are employed. Using a theoretical model that links statistical fluctuations to nonlinear light scattering responses, we demonstrate that in the unstable fluctuations phase (phase II, Figure 1A), the nanoscale interfacial solvent area surrounding the solute changes drastically, leading to changes in the SH intensity and, most notably, its standard deviation. By linking data to theory, essential insights into the solubilizing mechanism can be obtained, which are traced back to solute/solvent interactions. Exploiting the coherent nature of the SH emission together with the sensitivity to spatial arrangements creates a method that is exceptionally sensitive to small fluctuations in the interfacial area. This results in an enhanced detection limit of ∼1 nM, and simultaneously enables high-throughput measurements with a sustainability gain of >1000×. Compared with current industrial practice, this means a potential reduction of ∼3.5% of global CO_2_ emissions of the pharmaceutical industry is within reach.

## Experimental Methods

### Materials

Caffeine, tamoxifen, potassium hydroxide (KOH), albendazole (Sigma-Aldrich), 1,2-dimyristoyl-*sn*-glycero-3-phosphocholine (DMPC) in powder form (Avanti Lipids, Alabama), undecan-1-ol (C_11_H_240_, Sigma-Aldrich), dimethyl sulfoxide (DMSO, Sigma-Aldrich), and phosphate-buffered saline (PBS, Sigma-Aldrich) were used as received. Aqueous solutions were made with ultrapure water (H_2_O, Milli-Q UF plus, Millipore, Inc., electrical resistance of 18.2 MΩ cm). All aqueous solutions were filtered with 0.2 μM Millex filters.

### Cleaning Procedures

Glassware for sample preparation was cleaned with a Deconex (Borer Chemie AG) solution prepared by 1:20 dilution with ultrapure water (Milli-Q, Millipore, Inc., electrical resistance of 18.2 MΩ cm) and then rinsed thoroughly with ultrapure water at least 15 times.

### Sample Preparation

DMPC samples were obtained via serial dilution. The starting solution was prepared by weighing 650 μg of lipid powder in a 100 mL gauge flask. Ultrapure water was then added to complete the volume to 100 mL. The flask was sonicated afterward for 15 min. The obtained solution was then diluted two times in gauged flasks with sonication steps of 15 min to obtain a starting stock solution of 17.3 nM. From this starting stock solution, the serial dilution was done in 3 steps of 80%, followed by 9 steps of 90% around the expected critical micelle concentration (CMC) range, followed by 3 steps of 80% at lower concentrations. Typically, 1 mL was pipetted into a cylindrical glass sample cell for nonresonant second harmonic scattering measurement. An additional 9 (19 mL) of sample was removed from the gauged flask in the case of the 90 (80) % dilution steps. The volume of the sample in the gauged flask was then completed to 100 mL by adding 10 (20) mL of ultrapure water. The flask was then sonicated for 15 min before the next pipetting step for measurement and dilution. Undecanol samples were prepared via a serial dilution. The starting solution was prepared by pipetting 5.19 μL of undecanol into a 100 mL gauge flask. Ultrapure water was then added to complete the volume to 100 mL. The flask was sonicated afterward for 15 min to obtain a starting stock solution of 250 μM. From the starting stock solution, the serial dilution was done in 5 steps of 80%, followed by 6 steps of 90% around the expected solubility limit concentration. Typically, 1 mL was pipetted into a cylindrical glass sample cell for nonresonant second harmonic scattering measurement. An additional 9 mL (19 mL) of the sample was removed from the gauged flask in the case of the 90 (80) % dilution steps. The volume of sample in the gauge flask was then completed to 100 mL by adding 10 (20) mL of ultrapure water. The flask was then sonicated for 15 min before the next pipetting step for measurement and dilution.

Tamoxifen samples were prepared directly by weighing the powder in a flask and adding ultrapure water to reach the desired concentrations. Solubility measurements for albendazole samples were performed using the solvent shift method. From a stock solution of 10 mM albendazole in DMSO, a series of DMSO solutions was prepared by dilution in pure DMSO in the range 10 μM to 10 mM. From each of these stock solutions, a 1%, by volume, of the DMSO stock is transferred to a (filtered) PBS buffer (pH 7.4). The resulting mixture was centrifuged, sealed, and gently mixed in a well plate shaker for at least 2 h prior to measurements.

### Nonresonant Second Harmonic Scattering (SHS)

The setup used (Figure 1C) is described in ref 26. Briefly, 190 fs laser pulses centered at 1028 nm with a 200 kHz repetition rate are used to illuminate the sample. The polarization of input pulses was controlled by a Glan–Taylor polarizer (GT10-B, Thorlabs) in combination with a zero-order half-wave plate (WPH05M-1030). All measurements were conducted with input and output polarization set to P-polarized light, i.e., parallel to the horizontal plane of light scattering. The filtered (FEL0750, Thorlabs) input pulses with a pulse energy of 0.3 μJ (incident laser power *P* = 60 mW) were focused into a cylindrical glass sample cell (inner diameter 4.2 mm) with a waist diameter of ∼35 μm and a Rayleigh length of 0.94 mm. The scattered second harmonic light was collected with a plano-convex lens (*f* = 5 cm) and then filtered (ZET514/10×, Chroma), polarized (GT10-A, Thorlabs), and finally focused into a gated photomultiplier tube (H7421–40, Hamamatsu). The angle of acceptance for the signal collection was 3.4°. The scattering pattern was measured at a scanning step of 5°. Each data point was acquired with an acquisition time of 20 × 1 s and a gate width of 10 ns. All measurements were performed in a temperature- and humidity-controlled room (*T* = 297 K; relative humidity, 26.0%).

## Results and Discussion

### Coherent Intensity Fluctuations Are Linked to Nanoscale Interfacial Area and Solubility

Nonresonant elastic angle-resolved second harmonic scattering (SHS) requires the interaction of near-infrared optical fs laser pulses with a solution. Figure 1B shows the schematic of nonresonant second harmonic generation (SHG). SH photons are emitted when anisotropic molecules are distributed in an anisotropic manner.^28^ The different contrast mechanism and the difference between detection and illumination frequency make this method very different from linear light scattering: Coherent SHS originates from orientationally correlated molecules and is uniquely sensitive to the spatial symmetry/structure of the liquid medium. It is therefore sensitive to nanoscale interfaces,^29–31^ which emit scattered light in specific patterns.^32–36^ In such cases, SH light is primarily generated by the solvent molecules at the nanoscale interface. Besides the interfaces of nanoparticles, nonresonant coherent SH emission of solvent molecules was recorded from solutions containing pM amounts of polyelectrolytes,^37^ the adsorption of femtomolar amounts of protein on liposome interfaces in aqueous solution,^38^ and short-lived (∼500 fs) transient voids in neat water.^39^ These low detection limits were possible thanks to optimized laser pulse and detection parameters enabling a thousandfold increase in detection efficiency compared to state-of-the-art SHG instruments.^26,27^ In the case of linear light scattering, there are no selection rules that depend on spatial symmetries, and the contrast is proportional to the volume of solute weighted by the refractive index contrast between the solute and solvent.

The molecular solvent boundaries around solutes (I in Figure 1A) contain solvent molecules that are orientationally correlated on a nanometer scale, and they emit coherent SH photons having P-polarization. These photons emerge close to the forward direction of the SH scattering pattern, as explained in ref 39. As the concentration increases, aggregates form, which are initially unstable (II in Figure 1A, the dynamic aggregation regime). These aggregates become larger and more stable at higher monomer concentrations (III in Figure 1A). Between regions I and III, aggregates form and dissolve dynamically, and their nanoscale interfaces are formed and dissolved dynamically. This should result in an increase in the coherent interfacial solvent SH emission, which is likely also fluctuating in time when the monomer concentration is in region II.

To test these hypotheses, we examined the solubility of undecanol in water. Undecanol is listed as “insoluble” with an estimated solubility of ∼110 μM.^40,41^ Figure 1C shows a polar plot of the SH intensity of neat water (black), an undecanol solution below the solubility limit (region I, 50 μM, green), an undecanol mixture above the solubility limit (region II, 160 μM, blue). Figure 1C also shows a polar plot of the standard deviations (STDs) for the undecanol mixture at 160 μM (red, scaled by 1/2) and neat water (yellow). Figure 1D shows the time trace of the intensity of all three solutions measured at a scattering angle of 10°. The fundamental beam is indicated in Figure 1C, and θ = 0° marks the forward direction. The integration time of each data point was 1 s, with *N* = 20 measurements (see Experimental Methods). These data show an increase in SH intensity in the forward direction as well as a drastic change in the standard deviation. Performing similar recordings with linear light scattering does not yield similar results because the contrast mechanism is entirely different. A direct comparison of solubility measurements comparing second harmonic scattering and linear scattering is given in the SI, Section S2.

### Solvent Redistribution around Aggregates Leads to SHS Fluctuations

To understand the observations in Figure 1C,D, we theoretically describe the SH response from the solution, paying particular attention to the change in the coherent nanoscale interfacial solvent response as a function of solute concentration. The conceptual basis of this approach, the solvent redistribution (SR) method, is illustrated in Figure 2A. The model contains the number of bulk solute, solvent (here, water), and interfacial water molecules as ingredients, represented by *N*, *N_W_*, and *N_I_*, respectively, and their molecular hyperpolarizabilities (β_*S*_^(2)^, β_*W*_^(2)^, and β_I_^(2)^, respectively). In region II in Figure 1A, the amount of nanoscale interfacial solvent increases at the onset of aggregation, and there is a fluctuation in the nanoscale interfacial area. The change in SH intensity and its standard deviation can be used as a metric for solubility. Because we are interested in the coherent emission, which is best detected in the forward direction,^39^ the assumption is made that the difference in SH intensity from clusters of different sizes is solely due to the difference in the number of water molecules at the interface. The mean SH intensity is then

1 $$I\left(2\omega \right)=N_{W}{\left(\beta_{W}^{\left(2\right)}\right)}^{2}+N{\left(\beta_{S}^{\left(2\right)}\right)}^{2}+\left\langle N_{I}\right\rangle {\left(\beta_{I}^{\left(2\right)}\right)}^{2}$$

where ⟨*N*_*I*_⟩ represents the mean number of interfacial water/solvent molecules. The standard deviation (STD) is

2 $$\mathrm{STD}={\left(\left\langle N_{I}^{2}\right\rangle -{\left\langle N_{I}\right\rangle }^{2}\right)}^{1/2}{\left(\beta_{I}^{\left(2\right)}\right)}^{2}$$

STD are quantities that can both be described in terms of the statistics of the number of molecules at the interface. There are *N* solvent molecules in the system; *n*_*m*_ clusters with *m* constrained by ∑_*m* = 0_^*N*^ *mn*_*m*_ = *N*. The statistical mechanics of the system is best written in terms of the most probable number of clusters (*N*_*c*_), *N*_*c*_ = ∑_*m* = 1_^*N*^ *n*_*m*_. The canonical partition function can be written as^2^

3 $$Q=\sum_{n_{1}+\cdot \cdot \cdot +n_{N}=N_{C}}\left(\prod_{m=1}^{N}\frac{q_{m}^{n_{m}}}{n_{m}!}\right)=\frac{{\left(\sum_{m=1}^{N}q_{m}\right)}^{N_{C}}}{N_{C}!}$$

where *q*_*m*_ is the canonical partition function of a cluster with *m* molecules. The average number of interfacial molecules can now be expressed in terms of *Q*

4 $$\left\langle N_{I}\right\rangle =\frac{1}{Q}\sum_{n}\left[\sum_{m^{\prime }=1}^{N}{\left(m^{\prime }\right)}^{2/3}n_{m\prime }\right]\left(\prod_{m=1}^{N}\frac{q_{m}^{n_{m}}}{n_{m}!}\right)$$

with *m*′ being a dummy index. As worked out in Supporting Materials (S1), an expression can be found that links the SH intensity to the most probable nanoscale surface area and the fluctuations therein. This involves finding an expression for *N*_*c*_, and it is derived from constrained entropy maximization, which results in $N_{c}≅\sqrt{N/e}$ . Taking the thermodynamic limit, wherein the number of molecules in a cluster is treated as a continuous variable, and replacing the sums over *m* by integrals, the expressions for the SH intensity *I*(2ω) and its standard deviation STD from eqs 1 and 2 become

$$I\left(2\omega \right)=N_{w}{\left(\beta_{W}^{\left(2\right)}\right)}^{2}\left(2\omega \right)+N{\left(\beta_{S}^{\left(2\right)}\right)}^{2}\left(2\omega \right)+{\left(\frac{N}{e}\right)}^{1/2}{\left({\mathrm{\beta ̃}}_{I}^{\left(2\right)}\right)}^{2}\left(2\omega \right)\left(\frac{F_{2,N}}{F_{0,N}}\right)$$ 5

6 $$\mathrm{STD}={\left(\frac{N}{e}\right)}^{1/2}{\left[\left(\frac{F_{4,N}}{F_{0,N}}\right)-{\left(\frac{F_{2,N}}{F_{0,N}}\right)}^{2}\right]}^{1/2}{\left({\mathrm{\beta ̃}}_{I}^{\left(2\right)}\right)}^{2}\left(2\omega \right)$$

where ${\left({\mathrm{\beta ̃}}_{I}^{\left(2\right)}\right)}^{2}=\alpha {\left(\beta_{I}^{\left(2\right)}\right)}^{2}$ , where α is a constant, *F*_*n*,*N*_ are integral functions of the form $F_{n,N}=\int_{0}^{N}m^{n/3}q\left(m\right)dm$ , and *q*(*m*) is the partition function, which is given by *q*(*m*) = e^–β(μ(*N*)*m*+γ*m* ^*p*^)^ with *p* = 2/3 for a sphere and *p* = 1 for a rod-like aggregate. Here, β = 1/*kT*, with *k* the Boltzmann constant, and μ is the chemical potential or free energy, which we write as μ­(*N*) ≅ −μ̅_0_(*N* – *N*_0_), with μ̅_0_ = βμ_0_, and μ̅_0_ > 0. μ_0_ is the free energy difference between the bulk aggregate and the dissolved monomers. It describes how sharply the bulk free energy of an aggregate depends on the number of monomers inside it as the concentration gets close to the critical aggregation concentration (*c*_0_). *N*_0_ is the number of solvent monomers at the critical aggregation concentration. We also define the interfacial energy as γ̅ = βγ, which is the energy required to create a unit area of the solvent–solute interface. μ̅_0_ reports on monomer–monomer interactions, while γ̅ reports on the difference between interactions among monomers and monomer–solvent molecules. A large γ̅ means it is unfavorable to form an aggregate, while a large μ̅_0_ means it is easier to form an aggregate.

**Figure 2:**
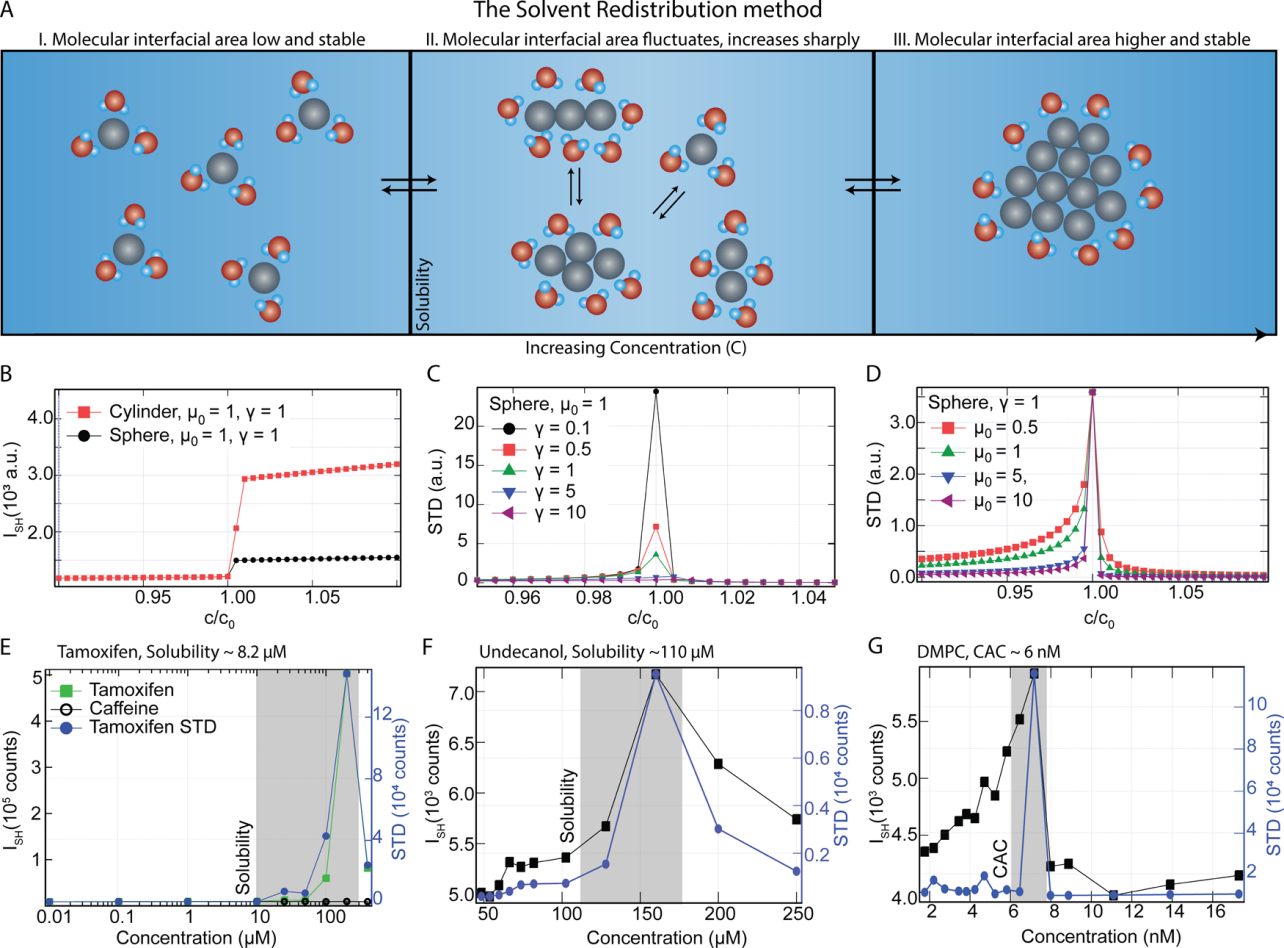
The Solvent Redistribution (SR) method, model, and experimental results. (A) Conceptual basis of the SR method, displaying the three stages toward aggregation, with emphasis on the surrounding solute, here represented as water. The monomers and aggregates have stable isotropic hydration shells. In the dynamic preaggregation phase, the structures of the hydration shells are changing, leading to an increasing amount of nanoscale interfacial area and standard deviation in the SH intensity. (B) Computed I_SH_ for cylindrical and spherical clusters as a function of concentration relative to the CAC. Both shapes display a drastic increase in the coherent I_SH_, as the nanoscale interfacial area increases. (C, D) STD as a function of concentration with varying interfacial energy γ (C), and bulk free energy of an aggregate close to the critical aggregation concentration, μ_0_ (D). (E–G) Solubility/aggregation measurements using the SR method for caffeine and tamoxifen (E). Caffeine is fully miscible with water in this concentration range.^42^ Tamoxifen is a drug with a reported kinetic solubility limit of ∼8.2 μM,^41^ and CAC of 191 μM,^42^ undecanol in water with a calculated solubility limit of 110 μM^39,40^ (F) and a phospholipid (dimyristoylphosphatidylcholine, DMPC) in water with a CAC of 6 nM (G, determined from fluorescence probe measurements^43^). The peak in the STD matches well with the reported CAC/solubility.

Figure 2B–E shows the computed SH intensity ([Fig fig2]B) and STD ([Fig fig2]C,D) of a system containing solute molecules with a critical aggregation concentration (CAC) c_0_. For *c* ≪ *c*_0_, the SH intensity increases linearly with *c*. This is expected, as there is a linear increase in the nanoscale solvent area when only monomers are present. When *c* ≃ *c*_0_, a sharp increase in the coherent SH intensity occurs, as the surface area of clusters increases and the number of interfacial water (solute) also increases (i.e., the third term of eq 5 dominates). When *c* > *c*_0_, solute molecules tend to form a single large cluster of *N* molecules, whose surface area is proportional to *N* ^2/3^. Increasing *c* generally leads to a continuing increase in the coherent SH intensity due to the increasing number of solute molecules and the increasing cluster surface area, but at a slower rate than in the concentration regime in which there are a large number of clusters of different sizes. Depending on the parameters, however, the intensity may also level off or decrease again (see SI, S1).

The standard deviation (STD) of the SHS intensity is shown in Figure 2C,D. The *STD* is small for *c* ≪ *c*_0_ and for *c* ≫ *c*_0_. For small concentrations, there are relatively few clusters and thus relatively few different sizes of cluster accessible, and most realizations of the system will have the same coherent scattering intensity. For very high concentrations, there is a single cluster containing all solute molecules, and all realizations will give the same scattering intensity. Thus, STD is small. However, when *c* ≃ *c*_0_, the STD is sharply peaked, as it becomes favorable for solute molecules to aggregate, the system can form a wide range of different clusters with a wide range of different sizes, and different realizations of the system will have very different intensities. Plotting the STD as a function of γ̅ (Figure 2C), a larger interfacial tension is correlated to a smaller STD. This can be explained in terms of the distribution in shapes/sizes, which is very limited for a large interfacial energy. The effect of μ̅_0_ is different: a high μ̅_0_ leads to a narrowing of the STD, in terms of concentration range, which is understood in terms of the sharpness of the aggregation transition that depends on μ̅_0_.

Based on this analysis, it is possible to determine the onset of aggregation by monitoring the solvent redistribution using coherent SH scattering in the forward direction (Figure 1C,D) and/or the standard deviation. Having μ̅_0_ and γ̅ as model inputs, together with two independent observables (*I*(2ω) and STD), it is in principle possible to understand the solubility process in the context of classical nucleation theory. Next, we apply the SR method to measure the solubility of several compounds in water: one that is fully miscible (caffeine), two with a solubility in the micromole range (tamoxifen and undecanol), and one insoluble phospholipid (1,2,-dimyristoyl-*sn*-glycero-3-phosphocholine, DMPC). More solvents/compounds can be found in the SI (S3, S5).

### SR Method, Practical Application, and Sustainable Solution

Figure 2E–G shows the SH intensity in the forward direction (left axis), together with the standard deviation (right axis) as a function of concentration for caffeine and tamoxifen (Figure 2E), undecanol (Figure 2F), and 1,2-dimyristoyl-*sn*-glycero-3-phosphocholine (DMPC, Figure 2G). The solubility value from the literature is indicated by the dashed lines and is 8.2 μM for tamoxifen using the dried DMSO method with 20 h incubation time,^4^ 110 μM for undecanol,^40,41^ and 6 nM for DMPC as determined from fluorescence probe measurements.^43,44^ It can be seen that for tamoxifen, undecanol, and DMPC, the SR method, and in particular the STD values, produce clear solubility values. Comparing the STD of undecanol to DMPC, undecanol has a broad STD range in terms of the concentration range, while the values for DMPC are much narrower. This can be understood by noting that undecanol forms micelles with the −OH groups facing the water. The interfacial tension and the free energy difference are rather low compared to DMPC, for which a spherical bilayer shell is formed, which is characterized by a larger difference in free energy. This matches the differences in the computed curves of Figure 2C,D.

Having a highly sensitive and reliable method for determining the solubility of compounds, a 96/384-well plate platform was implemented to enable high-throughput and small-volume (∼100 μL) measurements. This implementation, together with literature comparison to linear light scattering and HPLC-based detection, is described in the supporting materials (SI, S4) using a model drug, albendazole, as an example. The results of these and other measurements are combined in Table 1, which summarizes the current state-of-the-art methodologies for solubility measurements, comparing the sensitivity, reagent volume, and energy consumption for each method. The numbers are shown for an assumed consumption of 1000 96-well plates. HPLC-based methods, which are the current gold standard, suffer from excessive use of compounds, chemicals, and electricity,^45,46^ while linear light scattering (nephelometry) is economical but has poor sensitivity. The SR method enables a high-throughput format and reliable detection, provides potentially mechanistic information on the nature of the solubilization process, and reduces drastically the compound and chemical consumption. Given the ever-increasing scale on which solubility measurements are being performed, a worldwide switch of all solubility measurements to the SR method should lead to a potential reduction of ∼3.5% in the global CO_2_ emission of the pharmaceutical industry, a sustainability gain of >1000x (see SI S5 for the calculations).

**Table 1:** Comparison of Methods^a^,

method	process–contrast	detection Limit	reagent (L) and energy (kWh) use^b^
HPLC as prescreen	column separation + detection of extracted compound	<μM, if separation has gone well	2000 L (∼10000 kWh)
nephelometry	detection of fluctuations in the refractive index–differential measurement	∼20 μM; reproducibility issue	2 L (∼150 kWh)
SR method	fluctuations in nanoscale interfacial area, background-free (new wavelength)	∼nM	2 L (∼280 kWh)

a Methods, detection limit, reagent volume, and energy consumption for solubility screening, assuming 1000 96-well plates: The common R&D methods for solubility measurement are in the first two rows, the solvent redistribution method Is in the third row.

b Based on standard HPLC measurement: 2.5 min for column separation, 1.5 min to equilibrate and clean the columns with water/acetonitrile, flow of 1.3 mL/min, 3 reagents (water, acetonitrile, sample) for 5 min total, 6.5 mL per injection, electrical usage based on a measured LC-MS consumption of 1.33 kWh, supported ref [^44^].

## Conclusions

The pharmaceutical industry uses >12 million tons of chemicals annually,^6^ the majority of which is used for drug discovery. This quantity of chemicals requires a large amount of energy for their production, disposal, and recycling, contributing to 4.4% of global CO_2_ emissions, for which the pharmaceutical industry is responsible.^47^ A major contributor to this spending is high-throughput testing in drug discovery, and the biggest expenditure comes from solubility screening, a standard test that is required for every compound that is produced or tested. The current standard practice relies mostly on HPLC as a prescreening separation method, with a high sample consumption and a currently available detection limit of ∼1 μM. To meet the current trends of microbatch synthesis of specialized compounds and the need for smaller amounts in the nanotechnology sector, this sensitivity limit needs to be improved. Finally, current solubility/CAC measurements provide no mechanistic understanding of the process.

The SR method was devised to remediate these challenges that are currently inherent in the state-of-the-art practice. Exploiting the unique symmetry properties and sensitivity of high-throughput angle-resolved second harmonic scattering to the formation of nanoscale interfaces, an ∼1000x improvement on detection limit, waste prevention, and energy expenditure was achieved. A combination of classical nucleation theory with nonlinear light scattering was made, which predicted changes in the nanoscale solvent interfacial area prior to aggregation. This leads to (i) a leap in the coherent SH intensity and (ii) drastic fluctuations in the intensity, leading to a peaked STD, both of which appear at particular scattering angles only. The model also contains information that enables insight into the aggregation mechanism and shows that the expected behavior is independent of the solvent. SH experiments match the expectations of the model and show that a very high sensitivity of ∼<1 nM for the solubility limit is reached, with a minimal sample consumption and measurement time. Since the STD readout is simple and not influenced by background effects, a practical and easily implementable method for solubility/CAC measurements emerges. Upscaling the instrument with a 96/384-well plate format results in a reliable and sustainable method for determining solubility. We estimate that annually 70,000 t of chemical waste can be avoided, which means a reduction of 1.8 Mt of CO_2_ emissions. That is, a potential reduction of the pharmaceutical industry’s CO_2_ emissions by ∼3.5% is possible.

## Supplementary Material



## Notes

Second harmonic scattering during aggregation (S1); a comparison of second harmonic scattering and linear light scattering (S2); solubility measurements in other solvents: case of DMSO (S3); implementation of the solvent redistribution method in well plate format (S4); sustainability gains considerations (S5) (PDF)
