# Supplementary material for: Solvent Redistribution Method To Determine Solubility and Aggregation: High Throughput, Accuracy, and Sustainability
Source: J Phys Chem B. 2025 Aug 19;129(34):8798–805. doi: 10.1021/acs.jpcb.5c03073 (PMC12400408; doi:10.1021/acs.jpcb.5c03073)
Supplement: Supplementary file 1 [file jp5c03073_si_001.pdf]

Supporting Information

Solvent Redistribution Method To Determine Solubility And Aggregation: High throughput, Accuracy and Sustainability

O. B. Tarun[1#], N. Dupertuis[1#], David. M. Wilkins[2], S. Roke[1,3*]

[1]Laboratory for fundamental BioPhotonics, Institute of Bioengineering (IBI), School of Engineering (STI), École Polytechnique Fédérale de Lausanne (EPFL); CH-1015, Lausanne, Switzerland,
[2]Centre for Quantum Materials and Technologies, School of Mathematics and Physics, Queen's University Belfast, Belfast BT7 1NN, Northern Ireland, United Kingdom
[3]Institute of Materials Science and Engineering (IMX), School of Engineering (STI), École Polytechnique Fédérale de Lausanne (EPFL); CH-1015, Lausanne, Switzerland.
#both authors contributed equally
*sylvie.roke@epfl.ch

This PDF contains:

S1: Second harmonic scattering during aggregation
S2: Comparison of second harmonic scattering and linear light scattering
S3: Solubility measurements in other solvents: case of DMSO
S4: Implementation of the SR method in high throughput well-plate format
S5: Sustainability gains considerations

Figure S1: SHS intensity of a system of aggregates as a function of the concentration of solute monomers (normalized by the critical aggregation concentration c_0).
Figure S2: Standard deviation of the SHS intensity of a system of aggregates as a function of the concentration of solute monomers (normalized by the critical aggregation concentration c_0).
Figure S3: As Fig. S1 but with a shape-dependent contribution of the solute hyperpolarizability to the total intensity.
Figure S4: Comparison of second harmonic scattering and linear light scattering.
Figure S5: Solubility measurement with SR method using DMSO as solvent.
Figure S6: Implementation of the SR method to well-plate format.

S1. Second harmonic scattering during aggregation

Scattering Intensity and Aggregation

Consider a system with N solute molecules and N_W solvent (here, water) molecules and assume that the second-harmonic intensity from these types of scatterer add up incoherently. The solute molecules form aggregates, whose nature will be considered later on. The number of water molecules N_I at the nanoscale interface of solute aggregates is allowed to fluctuate. The SH scattering intensity from this system is,

$$I(2\omega; N_I) = N_W(\beta_W^{(2)})^2 + N(\beta_S^{(2)})^2 + N_I(\beta_I^{(2)})^2, \tag{1}$$

with $\beta_W^{(2)}$ a linear combination of second-order hyperpolarizability elements for a bulk water molecule, $\beta_S^{(2)}$ the same for a solute molecule and $\beta_I^{(2)}$ from an interfacial water molecule. The value of $\beta_W^{(2)}$ may be altered from that of a single water molecule to account for the fact that scattering from water is not purely incoherent [1].

Since the number of water and solute molecules is a constant, the average and standard deviation of the intensity are respectively,

$$I(2\omega) = N_W(\beta_W^{(2)})^2 + N(\beta_S^{(2)})^2 + \langle N_I \rangle (\beta_I^{(2)})^2, \tag{2}$$

$$\text{STD} = \left(\langle N_I^2 \rangle - \langle N_I \rangle^2\right)^{1/2} (\beta_I^{(2)})^2. \tag{3}$$

To evaluate these expressions, we use classical nucleation theory by Ford, in Ref. [2] to derive an expression for the average and standard deviation (STD) of the number of interfacial water molecules. We assume that the number of bulk water and solute molecules is known.

If the solute molecules form clusters, the instantaneous configuration of the system can be described using the vector $\boldsymbol{n} = (n_1, n_2, \ldots, n_N)$, where n_m is the number of clusters containing m solute molecules. This system has the constraint that, the number of molecules is fixed:

$$\sum_{m=0}^{N} m n_m = N. \tag{4}$$

Assuming that the clusters are independent of each other, the canonical partition function is,

$$Q = \sum_{\boldsymbol{n}} \left(\prod_{m=1}^{N} \frac{q_m^{n_m}}{n_m!} \right), \tag{5}$$

where q_m is the canonical partition function of a cluster with m molecules. The number of possible

vectors $\boldsymbol{n}$ commensurate with the constrained total number of solute molecules N is intractably large. We therefore proceed using the most probable number of clusters, N_C, and replace the sum over vectors $\boldsymbol{n}$ with a sum over all vectors such that $\sum_{m=1}^{N} n_m = N_C$. This is less restrictive than the standard method of most probable distributions in which the sum is replaced by the term that maximizes it [2]. It returns a summation that can be carried out using the multinomial theorem,

$$Q = \sum_{n_1+\cdots+n_N=N_C} \left(\prod_{m=1}^{N} \frac{q_m^{n_m}}{n_m!} \right) = \frac{\left(\sum_{m=1}^{N} q_m\right)^{N_C}}{N_C!}. \tag{6}$$

The average intensity and its standard deviation will thus be functions of the most probable number of clusters N_C, which will be considered further on.

Average and Standard Deviation of the Intensity

Expressions for the averaged SHS intensity and standard deviation, can be derived as a function of N_C, the most probable number of clusters. Assuming that clusters are approximately spherical, the surface area of a cluster with m molecules is proportional to $m^{2/3}$ and so $N_I \simeq a m^{2/3}$, where a is a constant. For simplicity, in the following we set,

$$(\tilde{\beta}_I^{(2)})^2 = a(\beta_I^{(2)})^2. \tag{7}$$

The average number of interfacial molecules is then,

$$\langle N_I \rangle = \frac{1}{Q} \sum_{\boldsymbol{n}} \left[\sum_{m'=1}^{N} (m')^{2/3} n_{m'} \right] \left(\prod_{m=1}^{N} \frac{q_m^{n_m}}{n_m!} \right). \tag{8}$$

We can make use of the standard statistical-mechanical trick of setting $q_m \to q_m e^{-\gamma m^{2/3}}$ whence,

$$\langle N_I \rangle = - \left. \frac{\partial \ln Q}{\partial \gamma} \right|_{\gamma=0}. \tag{9}$$

Combining this expression with Eq. (6) gives,

$$I(2\omega) = N_W(\beta_W^{(2)})^2 + N(\beta_S^{(2)})^2 + N_C(\tilde{\beta}_I^{(2)})^2 \left(\frac{\sum_{m=1}^{N} m^{2/3} q_m}{\sum_{m=1}^{N} q_m} \right), \tag{10}$$

and similarly,

$$\text{STD} = N_C \left[\left(\frac{\sum_{m=1}^{N} m^{4/3} q_m}{\sum_{m=1}^{N} q_m} \right) - \left(\frac{\sum_{m=1}^{N} m^{2/3} q_m}{\sum_{m=1}^{N} q_m} \right)^2 \right]^{1/2} (\tilde{\beta}_I^{(2)})^2. \tag{11}$$

To make these expressions more straightforward to integrate, we take the thermodynamic limit wherein

the number of molecules in a cluster is treated as a continuous, rather than a discrete variable, and replace the sums with integrals to give,

$$I(2\omega) = N_W(\beta_W^{(2)})^2 + N(\beta_S^{(2)})^2 + N_C(\tilde{\beta}_I^{(2)})^2 \left(\frac{\int_0^N m^{2/3} q(m)\, \mathrm{d}m}{\int_0^N q(m)\, \mathrm{d}m} \right), \tag{12}$$

$$\mathrm{STD} = N_C \left[\left(\frac{\int_0^N m^{4/3} q(m)\, \mathrm{d}m}{\int_0^N q(m)\, \mathrm{d}m} \right) - \left(\frac{\int_0^N m^{2/3} q(m)\, \mathrm{d}m}{\int_0^N q(m)\, \mathrm{d}m} \right)^2 \right]^{1/2} (\tilde{\beta}_I^{(2)})^2. \tag{13}$$

We will return to consider a model for the partition function $q(m)$ as a function of cluster size after finding an expression for N_C.

Most Probable Number of Clusters

The most probable number of clusters N_C is a function of the total number N of solute molecules. We can approximate the dependence of N_C on N using the principle of maximum entropy. The entropy of a system containing N solute molecules and where n_m is the number of clusters containing m molecules is,

$$S/k_\mathrm{B} = \frac{N!}{n_1! \cdots n_N!} \simeq N \ln N - \sum_{m=1}^{N} n_m \ln n_m, \tag{14}$$

using Stirling's approximation. We maximize this expression subject to the constraint that $\sum_{m=1}^{N} m n_m = N$, which is equivalent to maximizing the Lagrangian,

$$\mathcal{L} = -\sum_{m=1}^{N} \left[n_m \ln n_m + \lambda m n_m \right], \tag{15}$$

with λ an undetermined multiplier. To find the value of N_C that maximizes this Lagrangian, we find the vector $\boldsymbol{n}$ that maximizes $\mathcal{L}$: in doing so, we ignore the contribution of other vectors that give the same N_C, but in the thermodynamic limit these effects are small, as this is equivalent to the method of most probable distributions. This maximization leads to,

$$1 + \ln n_m + \lambda m = 0 \Rightarrow n_m = e^{-1-\lambda m}. \tag{16}$$

N_C, then becomes:

$$N_C = \sum_{m=1}^{N} n_m = e^{-1} \frac{1 - e^{-\lambda N}}{e^{\lambda} - 1}. \tag{17}$$

The parameter λ depends on N, and the dependence can be obtained by writing,

$$N = \sum_{m=1}^{N} m n_m = e^{-1} \frac{e^{\lambda} + N e^{-\lambda N} - (1+N) e^{-\lambda(N-1)}}{\left(e^{\lambda}-1\right)^2}, \tag{18}$$

which must be solved to find $\lambda = \lambda(N)$. As $N \to \infty$,

$$N \simeq e^{-1} \frac{e^{\lambda}}{(e^{\lambda}-1)^2}, \tag{19}$$

or,

$$\lambda = \pm \left(Ne\right)^{-1/2}. \tag{20}$$

Substituting this into Eq. (17) and taking the positive root to ensure that $N_C > 0$, we have,

$$\begin{aligned} N_C &\simeq e^{-1} \frac{1 - e^{-(N/e)^{1/2}}}{e^{(Ne)^{-1/2}} - 1}, \\ &\simeq \frac{e^{-1}}{e^{(Ne)^{-1/2}} - 1}, \\ &\simeq (N/e)^{1/2}. \end{aligned} \tag{21}$$

Inserting this expression into the formulae for the mean intensity and its standard deviation gives,

$$I(2\omega) = N_W (\beta_W^{(2)})^2 + N (\beta_S^{(2)})^2 + \left(\frac{N}{e}\right)^{1/2} (\tilde{\beta}_I^{(2)})^2 \left(\frac{\int_0^N m^{2/3} q(m)\,\mathrm{d}m}{\int_0^N q(m)\,\mathrm{d}m} \right), \tag{22}$$

$$\text{STD} = \left(\frac{N}{e}\right)^{1/2} \left[\left(\frac{\int_0^N m^{4/3} q(m)\,\mathrm{d}m}{\int_0^N q(m)\,\mathrm{d}m} \right) - \left(\frac{\int_0^N m^{2/3} q(m)\,\mathrm{d}m}{\int_0^N q(m)\,\mathrm{d}m} \right)^2 \right]^{1/2} (\tilde{\beta}_I^{(2)})^2. \tag{23}$$

Cluster Partition Function

To evaluate the average and standard deviation of the SHS intensity as in Eqs. (22), we require integrals of the form,

$$F_{n,N} = \int_0^N m^{n/3} q(m)\,\mathrm{d}m. \tag{24}$$

In classical nucleation theory the partition function $q(m)$ is given by [2],

$$q(m) = e^{-\left(\mu(N) m + \gamma m^{2/3}\right)/k_{\mathrm{B}} T}, \tag{25}$$

where $\mu(N)m$ is a bulk term such that $\mu(N) > 0$ for $N < N_0$ and $\mu(N) < 0$ for $N > N_0$, with N_0 the number of solute molecules corresponding to the critical aggregation concentration, and $\gamma m^{2/3}$ a surface term. With a change of variables $x = m^{1/3}$, proportional to the radius of the cluster we have,

$$F_{n,N} = 3 \int_0^{N^{1/3}} x^{n+2} e^{-\bar{\mu}(N)x^3 - \bar{\gamma}x^2} \, \mathrm{d}x, \tag{26}$$

where $\bar{\mu}(N) = \mu(N)/k_\mathrm{B}T$ and $\bar{\gamma} = \gamma/k_\mathrm{B}T$. Since we are interested in the behaviour of the intensity and standard deviation in the region of $N \simeq N_0$, we assume that the chemical potential $\bar{\mu}(N)$ behaves linearly in this neighbourhood,

$$\bar{\mu}(N) \simeq -\bar{\mu}_0 \left(N - N_0\right), \tag{27}$$

with $\bar{\mu}_0 > 0$. Given the parameters $\bar{\mu}_0$ and $\bar{\gamma}$ we can compute the $F_{n,N}$ numerically and the intensity and standard deviation are given by,

$$I(2\omega) = N_W(\beta_W^{(2)})^2 + N(\beta_S^{(2)})^2 + \left(\frac{N}{e}\right)^{1/2} (\tilde{\beta}_I^{(2)})^2 \left(\frac{F_{2,N}}{F_{0,N}}\right), \tag{28}$$

$$\text{STD} = \left(\frac{N}{e}\right)^{1/2} \left[\left(\frac{F_{4,N}}{F_{0,N}}\right) - \left(\frac{F_{2,N}}{F_{0,N}}\right)^2\right]^{1/2} (\tilde{\beta}_I^{(2)})^2. \tag{29}$$

Eq. (25) is the form appropriate for spherical clusters, in which the number of particles is proportional to the volume, $m \sim V \sim R^3$, with R the cluster radius, and the surface area $\sigma \sim R^2$, meaning that $\sigma \sim m^{2/3}$. For a cylindrical aggregate which grows along its axis, $\sigma \sim m$ and $q(m) = \exp\left(-(\mu(N)m + \gamma m)/k_\mathrm{B}T\right)$.

Behaviour of Intensity Average and Standard Deviation

Fig. S1 shows the SHS intensity of a system containing solute molecules with a concentration c_0. For concentrations $c \ll c_0$, the intensity has a simple linear increase with c (equivalent to a linear dependence on N) due to the second term in Eq. (28): that is, due to scattering from the solute molecules themselves. For $c \simeq c_0$, there is a sharp increase in the scattering intensity as the surface area of clusters increases and the number of interfacial waters also increases (i.e., the third term of Eq. (28) dominates). When $c \gg c_0$, it is overwhelmingly favourable for the solute molecules to form a single cluster of N molecules, whose surface area is proportional to $N^{2/3}$. The intensity will continue to increase, both due to the increasing number of solute molecules and the increasing cluster surface area, but at a slower rate than in the concentration regime in which there are a large number of clusters of different sizes.

The standard deviation of the SHS intensity is shown in Fig. S2: this quantity is relatively small for

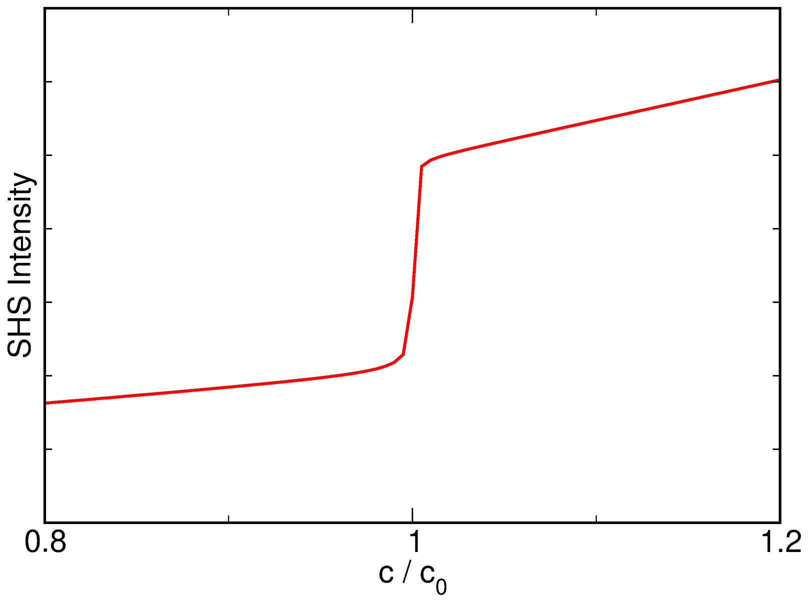

Figure S1: SHS intensity of a system of aggregates as a function of the concentration of solute monomers (normalized by the critical aggregation concentration c_0).

$c \ll c_0$ and for $c \gg c_0$, but has a peak at $c \simeq c_0$. For small concentrations, where there are relatively few clusters and thus relatively few different sizes of cluster accessible, most realizations of the system will have the same scattering intensity, while for very high concentrations there is a single cluster containing all solute molecules and all realizations will give the same scattering intensity. For $c \simeq c_0$, as it becomes favourable for solute molecules to aggregate, the system is able to form a wide range of different clusters with a wide range of different sizes, and different realizations of the system will have very different intensities.

To see the significance of the maximum in the standard deviation we write $\delta N = N - N_0$ and expand the standard deviation up to $\mathcal{O}(\delta N^2)$, taking $\bar{\gamma} = 0$ for simplicity. We find that the standard deviation achieves its maximum at $N^* = N_0 - c/N_0$, where c is a constant. That is, in the thermodynamic $N_0 \to \infty$ limit, the offset of this maximum from N_0 vanishes and the concentration at which the maximum is achieved is the critical aggregation concentration.

Dependence on Aggregate Shape

In deriving the intensity of Eq. (1), we assumed that the individual monomers scatter incoherently: this means that the scattering from the monomers depends only on the number N of monomers, and not on the shape in which they aggregate. In reality, the orientations of monomer molecules will be correlated

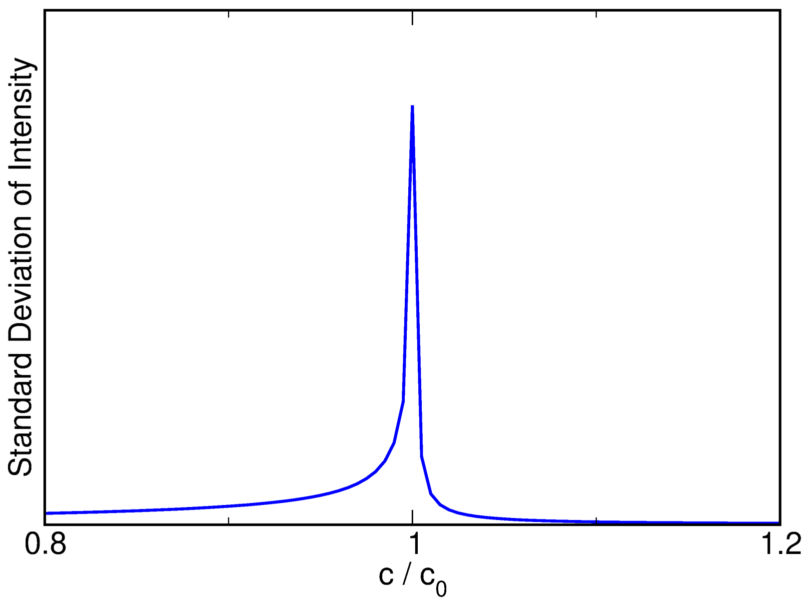

Figure S2: Standard deviation of the SHS intensity of a system of aggregates as a function of the concentration of solute monomers (normalized by the critical aggregation concentration c_0).

and,

$$I(2\omega; N_I) = N_W(\beta_W^{(2)})^2 + (\beta_S^{(2)}(N))^2 + N_I(\beta_I^{(2)})^2, \tag{30}$$

where $\beta_S^{(2)}(N)$ is the total contribution (coherent and incoherent) of the solute molecules to the scattering.

The dependence of the solute contribution on the number of solute molecules will be complex, and rather than attempting to derive this dependence in this paper, we suggest instead a phenomenological form for this function. For $N \ll N_0$, with few solute molecules and little aggregation, we expect that there will only be incoherent scattering from the solute molecules, giving the linear dependence of Eq. (1).

Limiting ourselves to $N \simeq N_0$ (equivalent to $c \simeq c_0$, close to the critical aggregation concentration), we note that for $N < N_0$ there is relatively little aggregation and the contribution to intensity will be largely incoherent, meaning that $\beta_S^2(N)$ increases roughly linearly with N. For $N > N_0$, the system will start to form larger aggregates; we expect these to be closer to spherical in shape, and thus more isotropic, meaning that $\beta_S^2(N)$ should decrease with increasing N. When $N \simeq N_0$, $(\beta_S^{(2)}(N))^2$ should reach a maximum, as the solute molecules are forming large numbers of aggregates with a wide variety of shapes. Overall,

$$(\beta_S^{(2)}(N))^2 \simeq \begin{cases} b_0 + b_-(N - N_0) & N < N_0, \\ b_0 + b_+(N_0 - N) & N > N_0, \end{cases} \tag{31}$$

where in general $b_+ \neq b_-$ but both constants are positive. Fig. S3 shows the dependence of intensity on the solute concentration for this type of dependence: following the critical aggregation concentration the SHS intensity decreases.

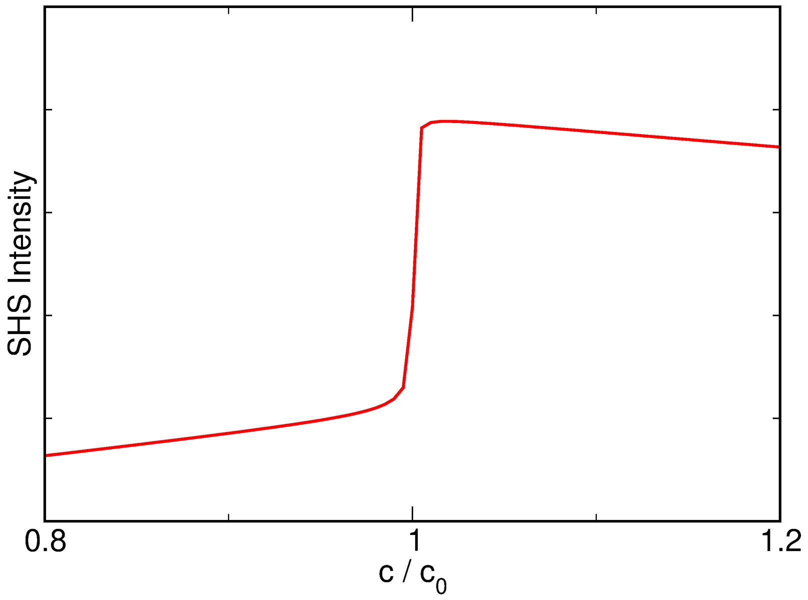

Figure S3: As Fig. S1 but with a shape-dependent contribution of the solute hyperpolarizability to the total intensity.

S2. Comparison of second harmonic scattering and linear light scattering.

The solubility limit of tamoxifen was measured by both the SR method as described in this work, and linear light scattering, using a standard nephelometry setup (BMG Labtech, nephelostar). Tamoxifen is a drug with a reported kinetic solubility limit of ~8.2 µM [3], and CAC of 191 µM [3]. The results are shown in Fig. S4. The linear light scattering (Fig. S4A) shows an increasing intensity (black triangles), and an increasing amount of fluctuations (STD, inverse open triangle Fig. S4A). The solubility limit was determined by segmented linear regression (two lines, one fitted to the baseline and the second line fitted to intensities above the baseline). The resulting calculated solubility limit is 51 µM. The change in intensity is coming from an increasing number of aggregates, proportional to the solute volume, and the square of the ratio of the refractive indices of the solute aggregate and the solvent.

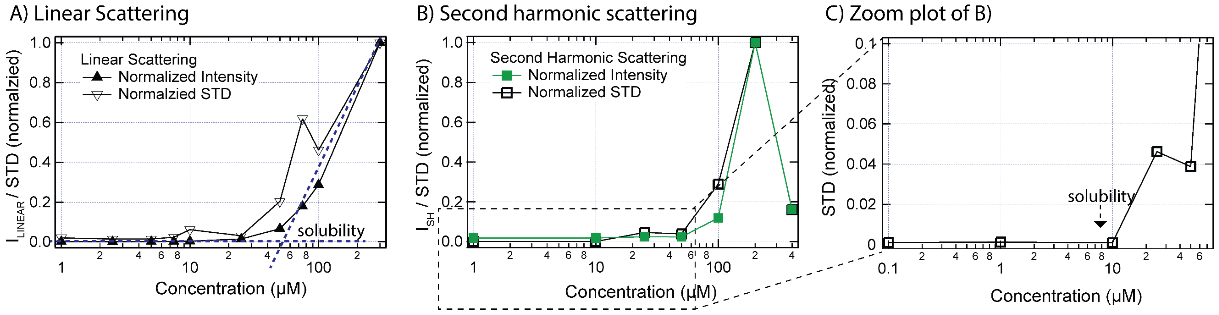

Figure S4: **Comparing second harmonic scattering and linear light scattering.** Comparison of (A) linear scattering and (B) second harmonic scattering (SHS) for the solubility measurement of tamoxifen in water and C) zoom plot B for the STD. The calculated solubility limit for linear scattering, based on segmented linear regression (dashed blue lines) is 51 µM. The solubility limit as seen by SHS is ~10 µM as shown in C). This is the concentration at which the STD starts to increase). The mean and STD of the SHS data were obtained from N=20 data points, while those for linear light scattering were obtained from 3 replicates.

The SH intensity recorded in the near-forward direction (green solid squares), and the STD (black open squares) are shown in Fig. S4B, Fig. S4C. Both the SHS intensity and the STD display a sharp increase followed by a drop, as described in the main text. The peak in the STD originates from an increase in the nanoscale interfacial area around unstable clusters, which leads to an increase as well as drastic fluctuations in the coherent SH intensity. This is followed by a stabilization in the interfacial area around aggregates that are stable (Fig. 1A, first region II, then region III). At concentrations beyond region II the instability is gone and the STD lowers again.

Thus, there is a clear difference in the emission / contrast mechanism of linear light scattering and coherent SHS, which is mainly due to the sensitivity of SHS to spatial symmetry,

which is absent for linear scattering. An additional difference comes from the fact that the SH emission occurs at a different wavelength from the fundamental beam, which creates a background free detection. For linear scattering emission and detection are done at the same wavelength, which means that the ratio is recorded, and discriminating small changes becomes difficult.

S3: Solubility measurements in other solvents: case of DMSO

Solubility measurements of potassium hydroxide (KOH) in dimethyl sulfoxide (DMSO) were performed using the solvent shift experiment. In this experiment, a series of concentrated KOH stock solutions in pure water were prepared. 1 vol% of one of these KOH-in-DMSO stock solutions was transferred to one of the wells of a 384-well plate containing liquid DMSO, with a total volume of 100 µL. The resulting mixture was centrifuged, sealed and gently mixed in a well-plate shaker for 24 hours prior to measurements. Figure S5 shows the results of the measurements using the SR method showing the intensity (Fig. S5A) and STD (Fig. S5B). The STD begins to increase at 2 mM, which matches the expected solubility of KOH dissolved in DMSO, as stated in Ref. [4].

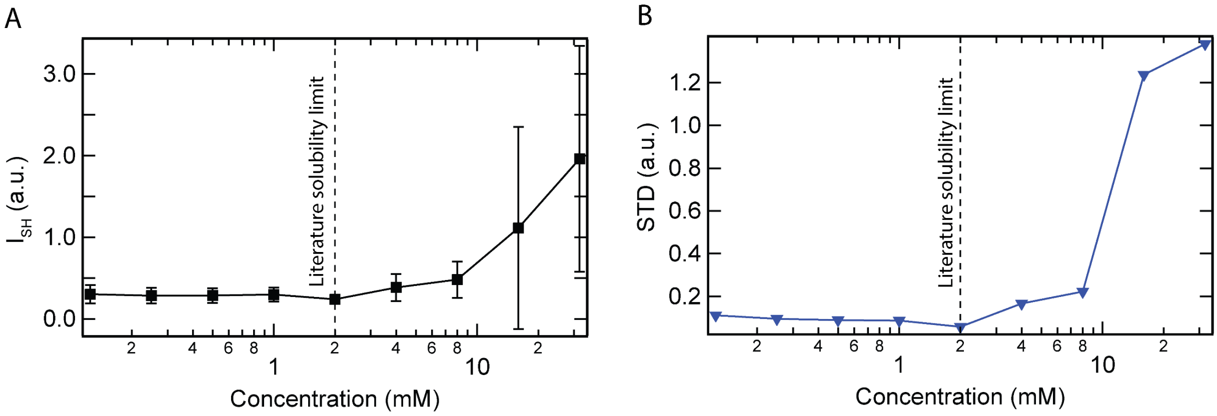

Figure S5: Solubility measurement with the SR method using DMSO as solvent. Solubility limit measurements for potassium hydroxide (KOH) in dimethyl sulfoxide (DMSO) using the solvent-shift experiment, **A**: intensity and **B**: STD plotted as a function of concentration. Both the intensity and standard deviation begin to increase at 2 mM, which agrees with the expected solubility limit of KOH in DMSO as reported in the literature in Ref. [4].

S4: Implementation of the SR method in high throughput well-plate format

In order to enable a higher throughput in a commercial / industrial setting a high-throughput device was constructed that uses a 96/384 well plate. Figure S6A shows an illustration of the setup for one example of an implementation of the SR method to high-throughput screening. An ultrafast laser with wavelength of 1030 nm, repetition rate of 200 kHz and pulse duration of ~210 fs is used to illuminate the sample (Carbide, Light Conversion). The laser beam is directed to a liquid sample contained in a standard 96/384 well-plate. A collection lens is used to collect the SH light at 515 nm. A set of optical filters consisting of a short pass filter and a bandpass filter were used to separate the SH beam from the fundamental. A collection lens was used to direct the scattered second harmonic intensities to a PMT detector (Hamamatsu).

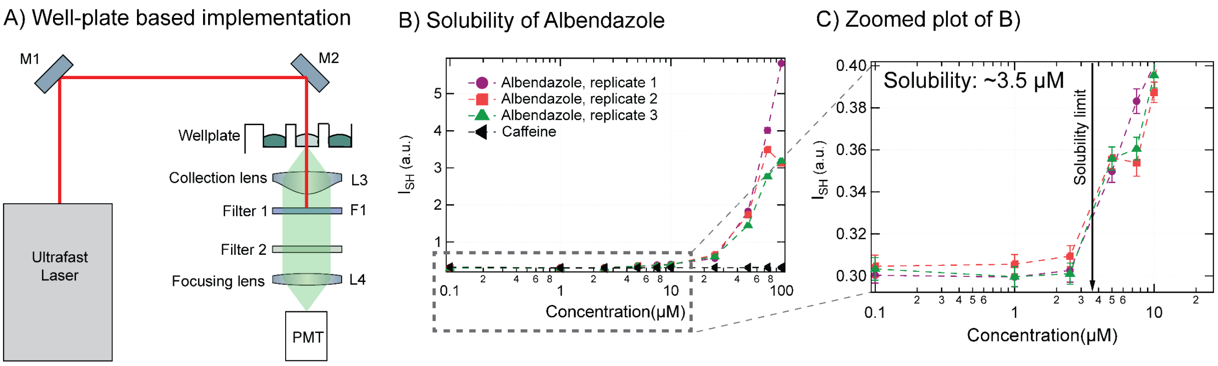

Figure S6. Implementation of the SR method to well-plate format. A: An ultrafast laser illuminates a liquid sample contained in a standard 96/384 well-plate from the top. A set of lenses and filters collects the scattered SH light and directs it to a photomultiplier tube (PMT). **B**: Example measurement using a model drug, Albendazole, with a measured kinetic solubility limit of 3.5 μM, three replicate of the same measurement are shown. The measurements are referenced caffeine, a soluble compound at the concentration range tested. **C)** Zoomed plot of the region of interest marked in B). The reported kinetic solubility limit for albendazole is between is 2.7 - 4.6 μM [5, 6].

Figure S6B and S6C shows kinetic solubility measurements for a model drug, albendazole (in triplicates), dissolved in aqueous PBS buffer solution (pH 7.4) using the solvent shift method. The measurements were performed with the instrument of Fig. S6A using the SR method. The measured kinetic solubility limit using the SR method is ~3.5 μM. This value lies within the range of reported values between 2.7 – 4.6 μM [5, 6].

S5. Sustainability gains considerations

Potential chemical and electricity savings of the SR method, with associated reduction in equivalent CO_2 emissions can be determined considering the difference measured between the SR method and a standard solubility measurement. A typical solubility measurement uses a separation step (filtration or chromatography/HPLC) in combination with detection, e.g. via mass spectroscopy (MS). HPLC and MS require large amounts of (i) electricity, to apply high pressure for the chromatography, and to ionize the sample in the mass spectrometer; (ii) chemicals (reagents and eluants) to operate the separation step and wash through the column; and (iii) consumables, such as separation columns, and well-plates for sample handling and filtration. Each of these consumption steps cost energy and lead to CO_2 emission. Electricity requires production and storage/transport to the site of the measurement. Chemicals (eluants and reagents, including deionized water) used in the measurement process have a carbon footprint during their lifecycle, from production to transport to recycling. And finally, the consumables themselves are a source of greenhouse gas emissions.

To determine the potential for more sustainability, we measured the consumption of chemicals and electricity for an HPLC-MS system (ThermoFisher system with Ultimate 3000 UHPLC coupled to MSQ plus Mass spectrometric detector) in the way it is used in solubility measurements (see also Ref. [7] from an HPLC manufacturer, Agilent, that considers the environmental impact of HPLC) and compared it to the SR method as performed by the instrument in Fig. S6A. The comparison was made during a period of 5 work days of operation. The savings are quantified per 96 well-plate. These numbers were then multiplied by an estimate of the number of well-plates used per drug discovery and development project, the number of instruments per company and the number of companies worldwide. The numbers are given in Table S1. In comparison with state-of-the-art HPLC-MS solubility setups, the SR method saves 6.5 mL per well, that is, 624 mL per 96 well-plate for each of three tested solvents: acetonitrile, deionized water, and formic acid. From this comparison we conclude that a worldwide deployment of the technology can save ~70 million liters or 70 kt of solvents per year.

Table S1: Total volume of chemicals saved if the SR method was used instead of HPLC-MS.

Mass of solvent saved per well (L)	Number of wells per plate	Number of plates per instrument per year	Instruments per company	Number of companies worldwide	Total volume of chemicals saved (millions of L)
0.0194	96	2000	2.5	7500	69.7

In terms of carbon footprint, the two main waste contributions are from chemicals and electricity. The three chemicals used in the measurement process are associated with equivalent CO_2 emissions (in kg of CO_2 per kg of solvent) [8, 9], as listed in Table S2.

Table S2: Equivalent CO_2 emissions for each solvent used in HPLC-MS.

	Volume of solvent used for the separation (mL)	Density (g/mL)	Equivalent CO_2 emissions (kg/kg of solvent)
Acetonitrile	6.5	0.8	3.5
Deionized water	6.5	1	0.8
Formic acid	6.5	1.22	2.51

The average CO_2 emissions for these three solvents is 2.22 kg/kg of solvent. A worldwide deployment of the SR method could therefore save ~0.16 Mt of CO_2 equivalent/year through chemicals savings only. In addition, the SR method with well-plate implementation saves electricity, by ~1.3 kWh per well (see Table 1 of main text) i.e. 124.8 kWh per 96 well-plate compared to the state-of-the-art. The reported CO_2 emissions in Europe and in the United States are respectively around 0.279 and 0.379 kg/kWh in 2022 (Table S3, [9, 10]).

Table S3: Equivalent CO_2 emissions from savings in electrical consumption compared to HPLC as pre-screen.

Electricity saved per well (kWh)	CO_2 emissions for electric consumption – EU & US (kg/kWh)	Number of wells per instrument	Instruments per company	Number of companies worldwide	Total savings in equivalent of CO_2 (Mt)
1.31	0.350	96*2000	2.5	7500	1.65

A worldwide deployment of the SR method could therefore save ~1.65 Mt of CO_2 equivalent/year through electricity savings. Chemical and electrical savings together amount to a total of ~1.80 Mt of CO_2 equivalent/year (including chemical savings). Note that this sustainability assessment does not include the consumables (columns, well-plates) necessary for sample preparation and measurement. Nevertheless, the order of magnitude of the impact on sustainability is clear. The pharmaceutical industry has an estimated 53 Mt of CO_2 emissions [11], which is 4.4 % of annual global CO_2 emissions. Implementing the SR method leads to a reduction of at least 3.5 % of CO_2 emissions.

References

(1) Tocci, G.; Liang, C.; Wilkins, D. M.; Roke, S.; Ceriotti, M. Second-Harmonic Scattering as a Probe of Structural Correlations in Liquids. *The Journal of Physical Chemistry Letters* **2016**, *7* (21), 4311-4316. DOI: 10.1021/acs.jpclett.6b01851.
(2) Ford, I. J. Nucleation theorems, the statistical mechanics of molecular clusters, and a revision of classical nucleation theory. *Physical Review E* **1997**, *56* (5), 5615-5629. DOI: 10.1103/physreve.56.5615.
(3) Ottaviani, G.; Wendelspiess, S.; Alvarez-Sánchez, R. Importance of Critical Micellar Concentration for the Prediction of Solubility Enhancement in Biorelevant Media. *Molecular Pharmaceutics* **2015**, *12* (4), 1171-1179. DOI: 10.1021/mp5006992.
(4) Dimethyl Sulfoxide (DMSO) Solubility Data, Bulletin # 102B Gaylord Chemical Company, L.L.C.: Bogalusa, LA 70427, 2007.
(5) Sou, T.; Bergstrom, C. A. S. Automated assays for thermodynamic (equilibrium) solubility determination. *Drug Discov Today Technol* **2018**, *27*, 11-19. DOI: 10.1016/j.ddtec.2018.04.004 From NLM Medline.
(6) Sugano, K.; Kato, T.; Suzuki, K.; Keiko, K.; Sujaku, T.; Mano, T. High throughput solubility measurement with automated polarized light microscopy analysis. *Journal of Pharmaceutical Sciences* **2006**, *95* (10), 2115-2122. DOI: 10.1002/jps.20628.
(7) *Do You Know the Environmental Impact of Your HPLC?* Agilent Technologies technical overview 2020. https://www.agilent.com/cs/library/technicaloverviews/public/technical-overview-hplc-environmental-impact-5994-2335en-agilent.pdf (accessed 2025 June 28).
(8) City of Winnipeg, C. *Emission factors in kg CO2-equivalent per unit*. 2012. https://legacy.winnipeg.ca/finance/findata/matmgt/documents/2012/682-2012/682-2012_appendix_h-wstp_south_end_plant_process_selection_report/appendix%207.pdf (accessed July 14, 2025).
(9) (IPCC), I. P. o. C. C. *IPCC Guidelines for National Greenhouse Gas Inventories. Volume 3: Industrial Processes and Product Use.* . 2006. https://www.ipcc-nggip.iges.or.jp/public/2006gl/ (accessed July 14, 2025).
(10) Hannah Ritchie, P. R. a. M. R. *"Data Page: Carbon intensity of electricity generation", part of the following publication: Hannah Ritchie, Pablo Rosado and Max Roser (2023) - "Energy". Data adapted from Ember, Energy Institute.* . 2023. https://ourworldindata.org/grapher/carbon-intensity-electricity (accessed July 14, 2025).
(11) World Economic, F. *How the pharmaceutical industry can reduce its climate impact*. 2022. https://www.weforum.org/stories/2022/11/pharmaceutical-industry-reduce-climate-impact/ (accessed July 14, 2025).
